# Biosimilars: Company Strategies to Capture Value from the Biologics Market 

**DOI:** 10.3390/ph5121393

**Published:** 2012-12-12

**Authors:** Bruno Calo-Fernández, Juan Leonardo Martínez-Hurtado

**Affiliations:** 1 Escuela Técnica Superior de Ingeniería, Universidad de Santiago de Compostela, Rúa Lope Gómez de Marzoa s/n, Campus Vida 15782, Santiago de Compostela, Spain; 2 Institute of Biotechnology, University of Cambridge, Tennis Court Rd, CB41QT, Cambridge, UK

**Keywords:** biosimilar, *patent cliff*, biopharmaceutical industry, market strategy, biologics

## Abstract

Patents for several biologic blockbusters will expire in the next few years. The arrival of *biosimilars*, the biologic equivalent of chemical generics, will have an impact on the current biopharmaceuticals market. Five core capabilities have been identified as paramount for those companies aiming to enter the biosimilars market: research and development, manufacturing, supporting activities, marketing, and lobbying. Understanding the importance of each of these capabilities will be key to maximising the value generated from the biologics *patent cliff*.

## 1. Introduction

**Figure 1:**
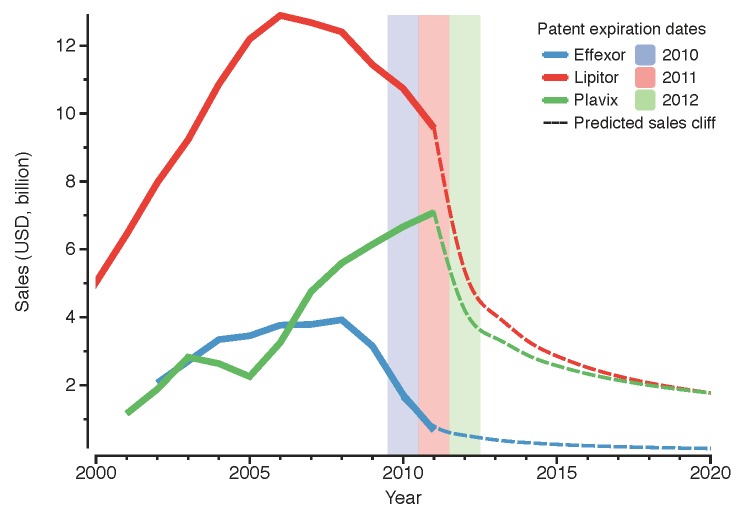
Global sales for three blockbuster chemical drugs: Effexor, Lipitor and Plavix. The solid coloured lines represent the annual sales of the product until 2011; the dotted lines represent a projection of the sales for the following years based on Effexor’s drop in revenue drop (*see* Appendix). The shadowed areas correspond to the patent expiration year as indicated in the legend (data from: [2,3]).

The term *patent cliff* refers to an abrupt drop in sales of a group of blockbuster (A blockbuster is defined as a medicine that exceeds global sales of 1 billion USD per annum [1].) products after reaching the end of their patent life. For example, Plavix, Singulair, Diovan and Lipitor are all chemical blockbuster drugs discovered in the 1990s, with patent expiration date falling between 2011 and 2015 [2,3,4,5]. Thus, the revenue obtained through the sales of these products is at risk of falling drastically within these years, contributing to the so-called chemical drug *patent cliff*. To further exemplify this, Figure 1 shows three chemical blockbusters: Effexor, Lipitor and Plavix, with patent expiration dates of 2010, 2011 and 2012 respectively [2,3]. As the figure shows, a decrease in global sales follows after the year of patent expiration. This is already evident for Effexor and Lipitor, and the same trend is expected for Plavix. The market implications of this *cliff* may impact key players in the biopharmaceutical industry positively or negatively. In this paper, we discuss a similar phenomenon related to biologic drugs: the upcoming biologic drug *patent cliff*. 

## 2. The Patent Cliff of Biologic Drugs

**Figure 2:**
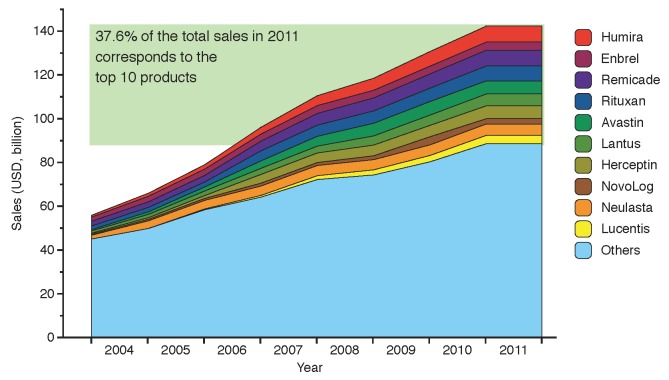
Evolution of global sales for the top ten branded biologic drugs from 2004 to 2011. The products below account for 37.6% of the total biologics market value, adding up to 53.4 billion USD in 2011 (data from financial statements 2004–2010 and [10,11,12,13,14,15]).

Therapies based on biologic products have been a disruptive innovation in the pharmaceutical industry due to their success in targeting previously unmet medical needs [6]. Sales of biologics reached 142 billion USD in 2011, equivalent to 19% of the global biopharmaceutical market in terms of revenue [7], with more than a third of this value (37.6%) captured by the top ten selling biologic products. The size of the market and its evolution from 2004 to 2011 is shown in Figure 2, highlighting the top ten selling brands: Humira, Enbrel, Remicade, Rituxan, Avastin, Lantus, Herceptin, NovoLog, Neulasta and Lucentis. 

**Figure 3:**
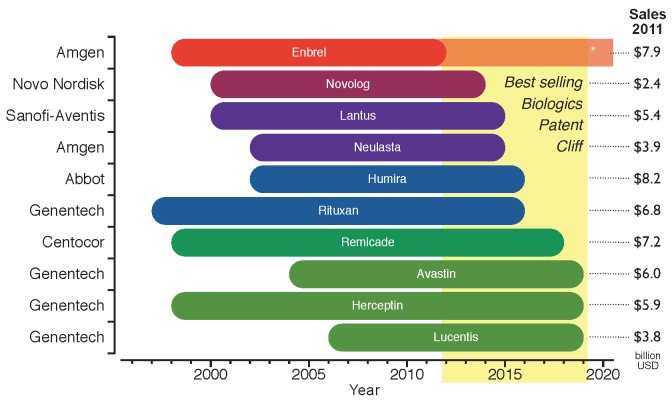
Period of market exclusivity for the top ten selling biologics. The 2012–2019 *patent cliff* is highlighted in yellow. The bars represent the period of market protection for each product from the approval date to the patent expiration date. Company names on the left hand side of the chart and total global sales for 2011 on the right hand side (data from: Financial Statements [10,11,12,13,14,15]). *Enbrel has been granted approval in 2011 for a patent filed in 1995, which competitors can still challenge [16].

There were two waves of biologic drug discoveries: recombinant versions of human endogenous molecules (*i.e.*, hormones and enzymes) were patented in the 1980s and more complex products, such as monoclonal antibodies, in the late 1990s [8,9]. Due to these patent protections and the high market prices of biologics, companies owning the IP have been generating substantial revenues (*see* Figure 2). However, this profitable period may not continue for long because of the proximity of the patent expiration dates. Figure 3 shows the period of market exclusivity for the top ten selling biologics in 2011, as well as the correspondent 53.4 billion USD of global sales at risk by product. The *patent cliff* for the top ten selling biologics spans the years from 2012 to 2019. Any drop in revenue will not be as pronounced as for chemical drugs, as will be discussed in Section 4. 

## 3. The Rise of the Biosimilars Market

The approval of new regulations in the biopharmaceutical industry, together with current global socioeconomics, technological innovations, and the *patent cliff* of biologics are the main drivers for the rise of the biosimilars market. 

### 3.1. Technological Innovations

Advances in DNA recombinant technology have improved the selection methods for high producing cell lines [17]. The introduction of less costly disposable bioreactors has contributed to a decrease in the idle time in manufacturing plants, thereby lowering production costs [18]. Moreover, the development of high binding efficiency matrices and their application in chromatography have eliminated bottlenecks in the downstream processes, increasing the annual output of manufacturing plants [19]. These advances in biomanufacturing, together with higher levels of protein expression, have improved production yields, reduced production times and lowered costs, increasing even further the profitability of biologics. However, access to these technologies is still a capability unique to those few players that have been investing through the years to built biologic manufacturing platforms. 

### 3.2. Global Socioeconomics

The impact of global recession on government budgets in recent years, especially in developed countries, is driving the implementation of cost control measures in national health systems, which are aiming to reduce expenditure in prescription drugs [20]. Subsidising less expensive copies of current biologics could be seen as an easy solution to reduce health care costs. On the other hand, the increase of disposable income in emerging markets, where some national health systems do not cover the costs of specialist care medicines, may increase the number of patients that can afford these products. However, in order to control the quality and efficacy of these inexpensive versions of biologics, it is necessary to introduce pathways to regulate their approval and commercialisation. 

### 3.3. Regulatory Initiatives

In 2006 the European Medical Agency (EMA) introduced a pathway to review and approve biologic equivalents based on commercially available biologics [21,22,23]. Following EMA’s success, the Biologics Price Competition and Innovation Act (BPCIA) of 2009 became law in the US, and the Food and Drug Administration (FDA) adopted a similar approach to the European body with regards to the commercialisation of these products. Thus, the concept of *biosimilar* was established [24]. Other countries including Canada, Japan and Korea have gradually adopted similar regulatory pathways [25]. 

These regulations, healthcare expenditure cuts and advances in manufacturing techniques have determined the appearance of a new market for biosimilars, which is comparable to that of generic drugs. However, the manufacturing processes for biosimilars are much more complex than the manufacturing processes for generics, leading to technical challenges that will have to be overcome. 

## 4. Complexities of Biomanufacturing and their Impact on the Exploitation of the Patent Cliff

The BPCIA defines *biosimilar* as “*a biological product highly similar to the reference product notwithstanding minor differences in clinically inactive components;” and with “no clinically meaningful differences between the biologic product and the reference product in terms of the safety, purity, and potency of the product.*” [24]. 

Biosimilars are therefore, fundamentally different to the generic versions of chemical drugs, not only because they are not a perfect copy of their originators, but also in terms of their size, molecular weight and three-dimensional structure. In fact, replicating the complex structure of biologics is what makes their manufacturing difficult. Biosimilars are manufactured using living cells, thereby introducing intrinsic variability into the production process. 

**Figure 4:**
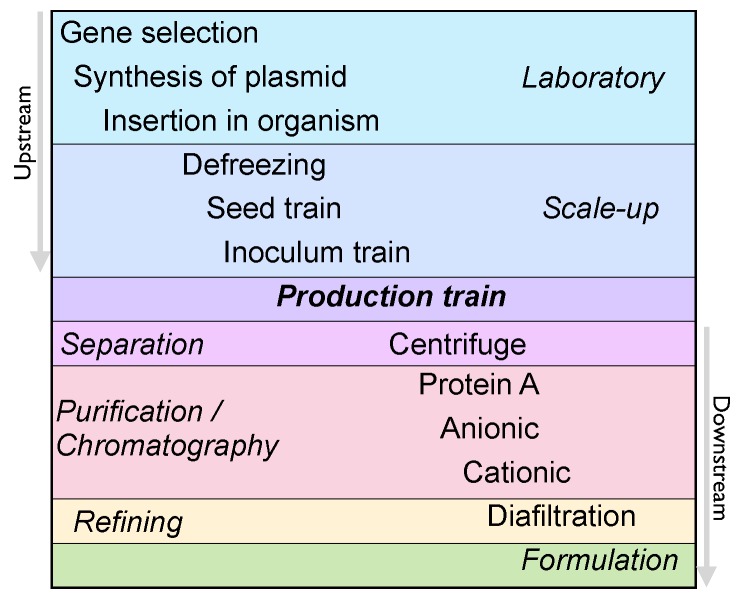
Typical steps of a manufacturing process for biologics. Upstream processes cover all the steps until the production stage and downstream processes cover the steps after it [30]. Even if the manufacturing steps are analogous between the biosimilar and the originator, their operation procedures may vary.

The manufacturing of biologics requires several stages of cell culture and purification, processes which are confidential to the company developing the product (*see* Figure 4). Therefore, as it is not possible for companies producing biosimilars to directly access this know-how, their manufacturing process will differ from that of its originators, and the structural variability of the product may be exacerbated. For example, different cell lines could affect the glycosilation and pegylation patterns [26]; oxidation and aggregation could also alter their three-dimensional structure [27]. These alterations can lead to catastrophic consequences for patient health, such as undesired immunogenic responses [28]. A famous, well-documented case of undesired immunogenicity was the death of nearly 150 patients in the US after the administration of biosimilar Heparin, an anticoagulant manufactured by Baxter [29]. 

Using product specific colorimetric assays (ELISAs) and size exclusion chromatography, it is possible to determine the molecular weight and size differences between biosimilars and innovator biologics; nuclear magnetic resonance or mass spectroscopy can be used to distinguish differences in their tertiary and quaternary structures; and gel electrophoresis and reverse phase high performance liquid chromatography are used to identify disparity in glycosylation patterns, aggregation and purity [31]. However, these techniques are not able to predict all biological activity in patients; even sophisticated *in vivo* models are not able to provide definitive conclusions regarding immunogenicity, because many immune responses are specie-specific [32]. 

Ultimately, only clinical trials (Phase I, II, III) and effective post-marketing pharmaco-vigilance (Phase IV) will ensure the same potency, safety and efficacy as the biologic originator [26]; Despite the ongoing academic debate, clinical trials are required by the regulatory agencies [33,34,35]. This requirement, together with the costly manufacturing processes, escalates the developing costs for biosimilars. It has been reported that the expected cost for their development could be between 75–250 million USD per molecule, one order of magnitude higher than the cost for generics [6,36,37]. 

Thus, the challenge for biosimilar manufacturers is not only to implement a robust manufacturing process from “scratch”, but also to prove comparability through clinical trials in a cost-effective manner. The high upfront costs needed, both to manufacture and to execute clinical trials will, therefore, act as a strong barrier to entry into the biosimilars market regardless of the *patent cliff*. Patent applications in the pipeline, non-patent exclusivity (e.g., orphan drug exclusivity, pediatric exclusivity, patent term extension/restoration, or new formulation exclusivity [38]) and litigation around them could also lead to delays. It is expected that the price drop for biosimilars will not be as pronounced as for generics, because companies entering the market need to overcome these barriers yet still make a financial return (it has been estimated that the market price for a biosimilar will be between 65% to 85% of its originator [39].) 

## 5. Key Capabilities for a Successful Entry to the Biosimilars Market

So far we have identified two capabilities necessary to enter and prevail in the biosimilars market: being able to fund basic research and develop clinical trials, and being able to access specific biomanufacturing platforms. Nonetheless, other capabilities are also required to ensure the successful introduction of the product into the market, these being: a global network of marketing sales representatives, legal expertise (e.g., patent litigation), and global distribution channels. Finally, lobbying with regulatory bodies, governments and opinion leaders may be required to accelerate the approval of legislation. This would encourage the substitution of currently prescribed patent-protected biologics and increase competition between biosimilars and their originators. 

Hence, the key capabilities are:

- Research and development
- Manufacturing
- Supporting activities: legal expertise and distribution channels
- Marketing
- Lobbying

The financial resources required to develop the products and to obtain these key capabilities will limit the number of players entering the biosimilars market to large companies with a strong financial position. However, not all these capabilities are required simultaneously, since this market is expected to evolve from “brand-driven” to “price-driven”. 

In the short term, it will be crucial for biosimilar companies to engage with healthcare professionals regarding the safety, potency and efficacy of biosimilars. Sales and market intake will be driven both by the brand of the product and the reputation of the company promoting it. Lobbying of healthcare institutions, governments and key opinion leaders, as well as accessing a global network of sales representatives, will therefore be necessary at this stage. 

In the mid-term, it is expected that all the actors of the biosimilar market, including regulatory bodies, national health systems, healthcare professionals and patients, will feel increasingly more confident about the prescription of biosimilars. Lobbying of regulatory authorities could encourage the approval of legislation supporting the substitution of biologics. Sales at this stage may be more price sensitive, encouraging competition between all the players in the biosimilars market. 

Eventually, in the long term, we can expect a mature biosimilars market showing similar dynamics to the current generics market, where sales are entirely driven by price. Therefore, generating economies of scale in manufacturing in order to lower production costs per unit, as well as acquiring global coverage through excellent distribution channels, will be the key capabilities required to succeed. 

The transition time between these three different stages will vary with the particularities of a geographical market, its generics culture and the strictness of its regulatory agencies. In order to cut healthcare expenditure, it will be a priority for both public and private payers to try to shorten this transition time, ultimately lowering the prices of biosimilars. 

**Figure 5:**
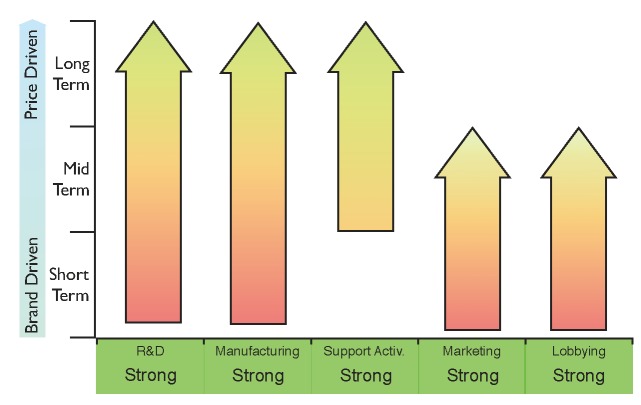
Proposed general strategy diagram for a successful entrance into the biosimilars market based on the five core capabilities identified. The requirement for key capabilities varies with the level of maturity of the market as indicated by the arrows: from brand-driven in the short term to price-driven in the long term.

Figure 5 shows the core capabilities that need to be reinforced at different stages of market maturity. This figure could be used as a general benchmark for companies entering the biosimilars arena, and could also help them to design their strategies to generate value from the upcoming *patent cliff*. 

## 6. The Current Scenario of the Biosimilar Industry

Innovative biotechs, generics companies, and *Big Pharma* have been attracted to the biosimilars market with the aim of fulfilling slightly different strategic objectives. *Big Pharma* are targeting biosimilars because they offer an easy option to diversify their product portfolio and create new revenue streams [40]. *Big Pharma* companies are characterised by “in-house” core capabilities in the research, development and marketing of chemical drugs. Moreover, their well-established industry lobbies are able to influence the opinion of government agencies, payers and regulators, which offers them an advantage in the biosimilars market [41]. Generics companies, on the other hand, have based their growth on the manufacture of chemical drugs at the lowest possible cost through economies of scale, on their expertise in patent litigation, and on their outstanding distribution channels [42]. Thus, the biosimilars market offers generics companies an opportunity to maximise the value of these supporting functions and also to diversify their portfolio of products. Innovative biotechs have developed core capabilities in the research, development and manufacturing of biologic products “in-house”. For them, the biosimilars market is an alternative method of generating revenue streams to reinvest in research. It is also a method of maximising the utilisation of their development and manufacturing capabilities, while at the same time undermining the portfolios of competitors. In the following sections, we analyse biosimilars market entry examples of *Big Pharma*, generic companies and innovative biotechs, based on the five key capabilities previously identified. 

### 6.1. The Case of Big Pharma

*Big Pharma* were mere spectators of the success of innovative biotech companies in the late 1990s, but they have recently acquired some of those successful biotechs to amend their lack of biologic capabilities [43]. The mega-mergers of Pfizer and Wyeth, Roche and Genentech, and Merck and Schering-Plough are the best examples of these acquisitions [44,45]. However, innovations in biologics are not as numerous as they were in the late 1990s, and the growth of the biologics market has started to slow down [46,47]. There are two main reasons for this deceleration: innovative biotechs had patented and developed products saturating the currently available approved indications, and regulatory agencies require new products to show better efficacy than the existing ones [46,47,48]. It has been suggested that pharma mega-mergers have been value destructive for *Big Pharma* [45]. It is therefore necessary for them to find alternative ways of creating value from these acquisitions, and entering the biosimilars market may be one of them. 

**Figure 6:**
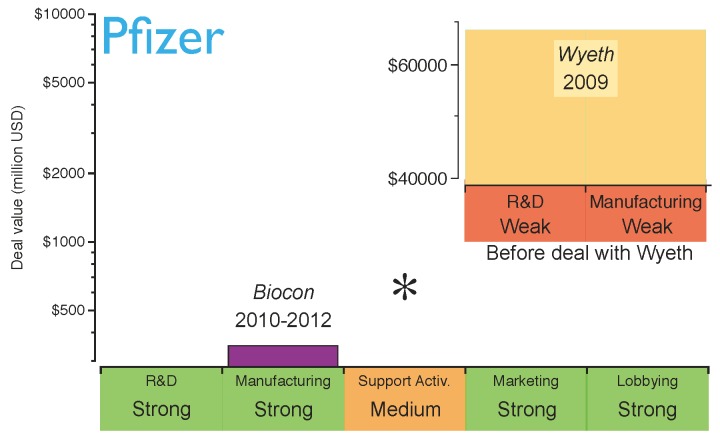
Pfizer’s capabilities for the biosimilars market after establishing the strategic deals. The capabilities have been graded as strong (green), medium (orange) or weak (red) based on the company’s profile before the deals. The asterisk indicates the capabilities the company should reinforce to gain competitive advantage. Shown in the inserted subfigure are Pfizer’s capabilities in biosimilars before the acquisition of Wyeth and the contributions of this deal.

An example of this is Pfizer, which in 2009 acquired Wyeth, one of the largest innovative biotechs, accessing its research, development and manufacturing platforms [49]. At that time, this deal was done with the objective of competing in the production and commercialisation of innovative biologics. Badly affected by the current chemical *patent cliff* (*see* drop in revenue of Lipitor and Effexor in Figure 1), Pfizer also stepped into the biosimilars market, establishing a collaboration with Biocon to produce biosimilar insulin in 2010 [50]. Biocon provided the manufacturing expertise and Pfizer was responsible for the sales strategy, thereby promoting biosimilar insulin and creating synergies within its diabetes portfolio. However, two years later Pfizer came to recognise that the largest financial returns were not in the insulin market. Consequently, it terminated its collaboration with Biocon. Pfizer is now planning to focus on the development of biosimilar monoclonal antibodies, which require more complex manufacturing processes than insulin, thereby making full utilisation of their “in-house” capabilities obtained through the Wyeth acquisition. Therefore, the capabilities obtained through Wyeth are now helping Pfizer to consolidate its biosimilars strategy [49]. Pfizer has recently started Phase I clinical trials for a biosimilar version of the monoclonal antibody Rituxan [51]. This is a good example of generating extra value from acquisitions for research and manufacturing biologics. The evolution of Pfizer’s strategy is shown in Figure 6; the figure highlights the strength of its key capabilities before the strategic deal with Biocon. 

Based on Pfizer’s current position, it can be concluded that the company has acquired capabilities for a rapid market intake of biosimilars in the short term. However, as the biosimilars market matures and evolves from brand-driven to price-driven, Pfizer may need to strengthen its supporting activities in the long term (*i.e.*, distribution channels). 

### 6.2. The Case of Generics Companies

Companies such as Teva, Sandoz and Hospira, the largest generics companies, are already commercialising biosimilar hormones, cytokines, and enzymes (e.g., insulin, EPO, interferon, G-CSF and imiglucerase) [5,52,53]. The manufacturing processes of these well-characterised endogenous molecules are not as complex as those for monoclonal antibodies [30,54]. However, generics producers have acquired invaluable experience from their early move into the biosimilars market, generating a substantial financial return to step into the higher value opportunity of biosimilar monoclonal antibodies. 

**Figure 7:**
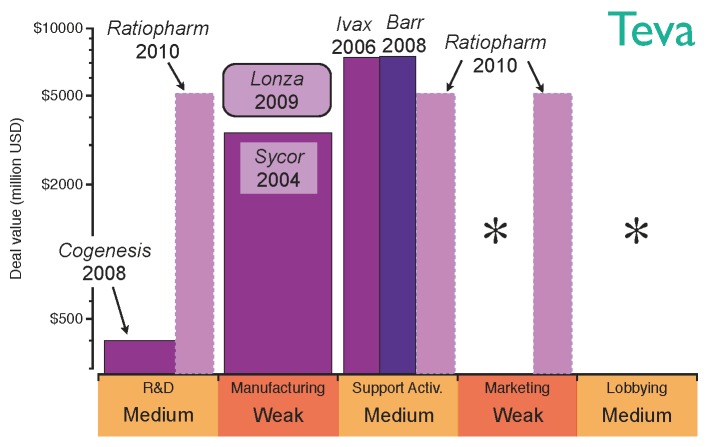
Teva’s capabilities for the biosimilars market after establishing the strategic deals. The capabilities have been graded as strong (green), medium (orange) or weak (red) based on the company’s profile before the deals. The asterisks indicate the capabilities the company should reinforce to gain competitive advantage.

The strategy of Teva, the largest generics company by revenue, reflects this transition from well-characterised biosimilar molecules to higher-value opportunities. Teva’s first step into biosimilars was the acquisition of Sicor in 2004, which provided a platform for the manufacture of hormones and cytokines [55]. At that time, there were still no regulations for the approval of biosimilars in Europe and the US, the two largest biopharmaceutical markets. However, after EMA approved a regulatory pathway for biosimilars in 2006, this early acquisition of Sicor enabled Teva to enjoy a competitive advantage. A further strategic move to commercialise its generics products globally was the acquisition of Ivax in 2006 [56]; this acquisition provided stronger distribution channels in Central and Eastern Europe in the same year that biosimilar regulatory pathways were approved by the EMA [22,23]. Teva’s position on Latin America, where local agencies were due to approve regulatory pathways, was also reinforced with this deal [57,58]. Distribution channels in Europe and US were further strengthened by the acquisition of Barr in 2008. Pliva, Barr’s Croatian subsidiary, granted Teva additional capabilities to develop biosmilar erythropoietin and granulocyte colony-stimulating factor [59]. That year, CoGenesys was also acquired, adding manufacturing facilities for peptide and protein-based drugs. Following those acquisitions, Teva established a joint venture with Lonza Biologics in 2009 to manufacture monoclonal antibodies and access higher value opportunities [60]. By this point in time, Teva had built a complete platform to develop and manufacture biosimilars for the top selling biologics, the patent lives of which come to their end in the next years. However, Teva’s appetite for acquisitions has not been interrupted; in 2010 the company purchased German Ratiopharm, thus strengthening its R&D capabilities and filling gaps in its European distribution channels [61]. Nevertheless, Teva’s global marketing may still need improvement; the evolution of Teva’s strategy in the biosimilar market and its acquisition of capabilities are shown in Figure 7. 

Teva’s approach to biosimilars was based on acquisitions rather than organic growth, obtaining immediate access to strong R&D, manufacturing and distribution channels; this acquisition strategy has rapidly increased not only Teva’s size, but also its lobbying power. Nevertheless, strengthening its marketing position will be key if the company wants to engage with healthcare professionals and boost the prescriptions of its biosimilars portfolio. This lack of marketing capabilities is inherited from its generics business model. However, its excellent distribution channels and patent litigation expertise put Teva in a favourable position to compete in the price-driven biosimilars market in the long term. 

### 6.3. The Case of Innovative Biotechs

Established innovative biotech companies fund their R&D operations through the revenues obtained from their biologic blockbusters, the majority of which were patented during the wave of biologic drug discoveries of the late 1990s [9]. The strategic decisions of generics companies and *Big Pharma* entering the biosimilar market are therefore a real threat for the survival of innovative biotechs. 

Entering the biosimilar market may seem to be a discordant strategy for biotechnological companies focused on the discovery and development of innovative products. However, their research, manufacturing and marketing capabilities would allow them to produce and commercialise biosimilars at virtually no extra cost and, on the other hand, it would offer them an opportunity to generate extra financial resources for their R&D operations. Logically, established biotechs will not develop biosimilars of their own biologics portfolio; they may, however, target the portfolios of their competitors to undermine their sales and weaken their financial position. 

**Figure 8:**
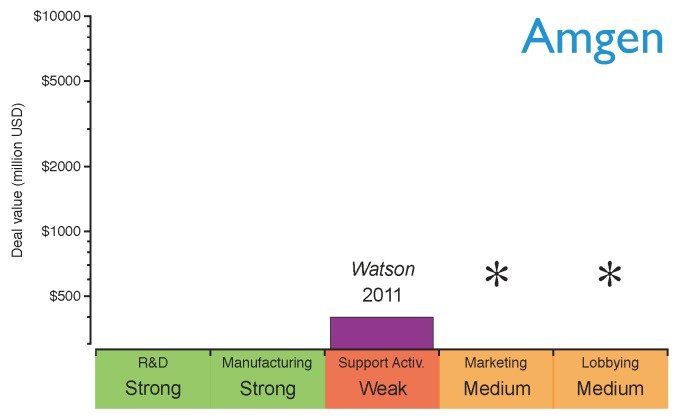
Amgen’s capabilities for the biosimilars market after establishing the strategic deals. The capabilities have been graded as strong (green), medium (orange) or weak (red) based on the company’s profile before the deals. The asterisks indicate the capabilities the company should reinforce to gain competitive advantage.

Amgen, one of the largest established innovative biotechs, has recently announced its plans to produce biosimilars driven by the proximity of the patent expiration of its old biologics portfolio (*see* Figure 3) and its recent lack of success in developing new blockbusters [10,62]. However, Amgen is not entering the biosimilars market alone; a collaboration agreement with the generics giant Watson was signed to develop biosimilar monoclonal antibodies [10,62]. Through this collaboration, Amgen will provide the R&D and manufacturing capabilities to produce biologics, and Watson will offer its expertise in supporting activities to compete in a price-driven market scenario. Watson will also contribute financially by improving Amgen’s cash position, and also by sharing the costs of developing the products. Amgen’s strategy for entering the market is shown in Figure 8. As can be seen, Amgen will need to build stronger marketing and lobbying capabilities in order to obtain a rapid market intake in the short term. 

Comparing the general strategy diagram of Figure 5 with the current capabilities of Amgen, Teva and Pfizer, it is possible to identify how their strategic deals have strengthened their position with regard to entering the biosimilars market. The capabilities marked with asterisks in Figure 6, Figure 7 and Figure 8 still need to be reinforced to acquire competitive advantage at different stages of maturity of the market. It cannot be inferred that the strategy for these specific examples will apply to any other *Big Pharma*, generics company or innovative biotech. Nevertheless, benchmarking the current capabilities of a company against the diagram in Figure 5 may give a good indication of which capabilities need to be reinforced. 

## 7. Conclusions

Innovative biotechnological companies have been enjoying bonanza times since the biodrug discoveries of the 1990s. However, the market value captured by these companies is under threat due to the imminent biologics *patent cliff*. This *patent cliff*, together with public healthcare budget cuts, technological innovations for manufacturing biologics and the approval of new regulations in the biopharmaceutical industry, has driven the consolidation of the biosimilars market. *Big Pharma*, generics companies and innovative biotechs have already positioned themselves to enter this market by acquiring or developing R&D, manufacturing, supporting activities, marketing or lobbying capabilities. Understanding the dynamics of the biosimilars market will be key for those companies aiming to pursue opportunities in biosimilars. The general strategy diagram proposed in this paper can be used as an accurate capability-benchmarking tool for any biopharmaceutical company planning to generate value from the biologics *patent cliff*. 

## Figures and Tables

**Figure A1:**
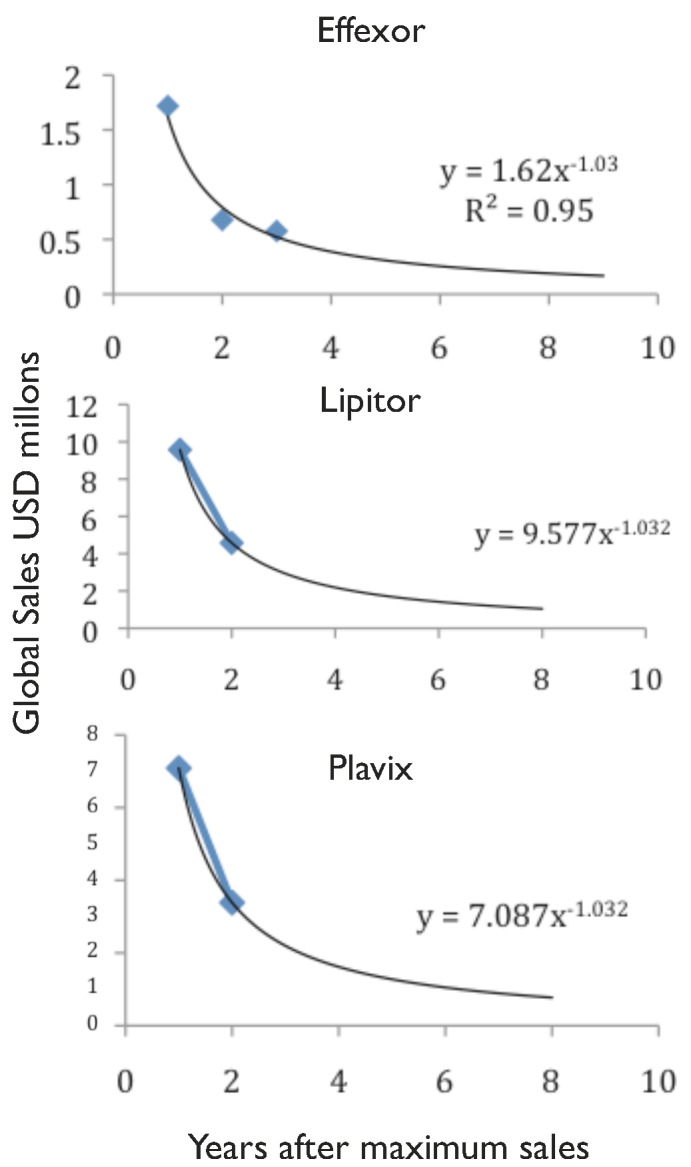
Sales forecast for Figure 1.

## Appendix

The sales decay forecast for Figure 1 was produced with an exponential model based on the sales for Effexor, which patent expiration date is 2011. The data was extracted from the 10-K Financial Statements. Year one corresponds to the year of sales year before the patent expiration. The forecast equation is:

*Sales* = *A* * (years after peak sales)*^B^*

where *A* is the peak sales value and *B* the exponential coefficient ≈ (−1.0). The projections are shown in annexed Figure A1.
